# Supplementary material for: Silkworm model of biofilm formation: In vivo evaluation of antimicrobial tolerance of a cross-kingdom dual-species (Escherichia coli and Candida albicans) biofilm on catheter material
Source: PLoS One. 2023 Jul 14;18(7):e0288452. doi: 10.1371/journal.pone.0288452 (PMC10348565; doi:10.1371/journal.pone.0288452)
Supplement: S1 File — (PDF) [file pone.0288452.s001.pdf]

Silkworm model of biofilm formation: *In vivo* evaluation of antimicrobial tolerance of a cross-kingdom dual-species (*Escherichia coli* and *Candida albicans*) biofilm on catheter material

Shintaro Eshima[1], Yasuhiko Matsumoto[1,*], Sanae Kurakado[1], Takashi Sugita[1]

[1]Department of Microbiology, Meiji Pharmaceutical University, 2-522-1, Noshio, Kiyose, Tokyo 204-8588, Japan.

*Corresponding author

E-mail: ymatsumoto@my-pharm.ac.jp (YM)

Table S1 MIC values of antibacterial drugs against *E. coli* RB-3 strain

Antibacterial drugs	MIC value[a] (µg/mL)
Meropenem	< 1
Levofloxacin	< 0.5
Ceftriaxone	< 1
Cefmetazole	< 8

[a]The MIC values of antibacterial drugs against *E. coli* RB-3. Susceptibility testing for the antibacterial drugs was performed using the MicroScan AST panel (Beckman Coulter, Pasadena,g CA, USA) according to CLSI M100-Ed31 (Clinical and Laboratory Standards Institute (CLSI). Performance standards for antimicrobial susceptibility testing; M100-Ed30. 30th ed. Wayne, PA: CLSI; 2020.).

Table S2 MIC values of antifungal drugs against *C. albicans* SC5314 strain

Antibacterial drugs	MIC value[a] (μg/mL)
Micafungin	0.03
Caspofungin	0.5

[a]The MIC values of antifungal drugs against *C. albicans* SC5314. Antifungal susceptibility testing was performed using a dry plate for antifungal susceptibility testing of yeasts (Eiken Chemical Co., Ltd., Tokyo, Japan) based on CLSI M27-A3. The MICs for micafungin and caspofungin were determined as the lowest concentration at which turbidity reached a 50% reduction compared with the positive control (IC_{50}).

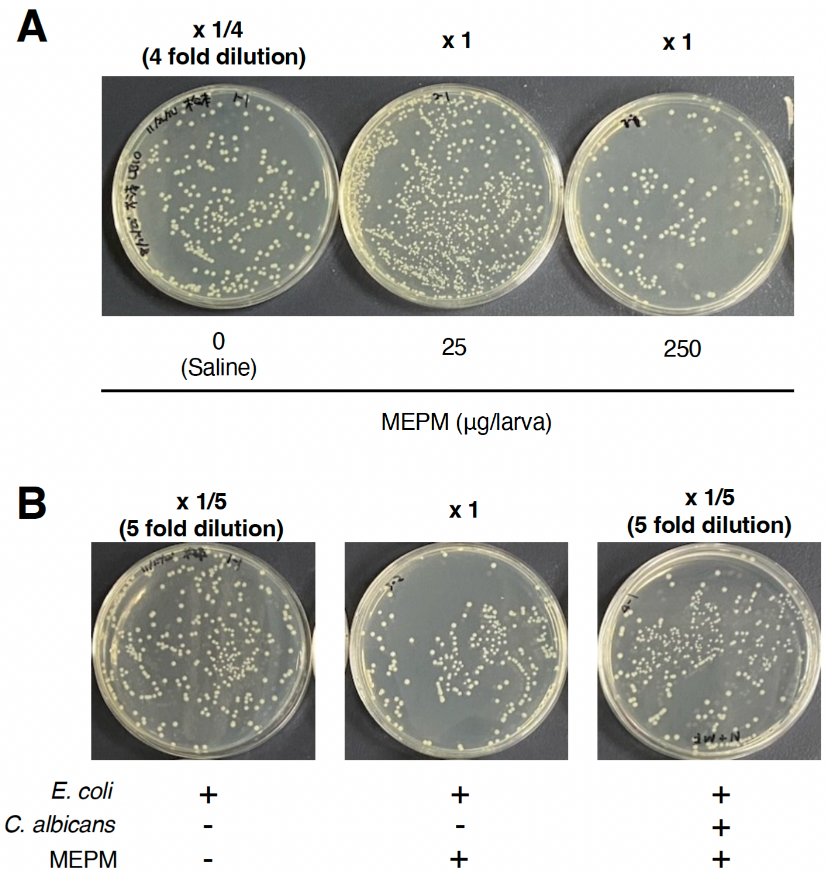

Supplementary Fig. S1 Plate images of viable *E. coli* cells in the biofilm on the surface of the PFs in the silkworms.

(**A**) Plate images of viable *E. coli* cells on the surface of the PFs in the silkworms administered MEPM (0–250 µg/50 µL) were shown. *E. coli* cell suspension (2 x 10^8 cells/50 µL) was inoculated into PF-inserted silkworms, and the infected silkworms were incubated at 27°C for 18 h. After incubation, saline or MEPM solution (0–250 µg/50 µL) was administered, and the silkworms were incubated at 27°C for 1 h. Viable *E. coli* cells on the surface of the PFs in the silkworms were grown on nutrient agar medium. (**B**) Plate images of viable *E. coli* cells on the surface of the PFs in the silkworms administered MEPM (0–250 µg/50 µL) were shown. *E. coli* cell suspension (2 x 10^8 cells/50 µL) or a mixed cell suspension (*E. coli*: 2 x 10^8 cells and *C. albicans*: 1 x 10^6 cells/50 µL) was inoculated into PF-inserted silkworms, and the infected silkworms were incubated at 27°C for 18 h. After incubation, saline or MEPM solution (0 or 250 µg/50 µL) was administered, and the silkworms were incubated at 27°C for 1 h. Viable *E. coli* cells on the surface of the PFs in the silkworms were grown on nutrient agar medium containing micafungin (1 µg/mL).
