# Supplementary material for: Silkworm model of biofilm formation: In vivo evaluation of antimicrobial tolerance of a cross-kingdom dual-species (Escherichia coli and Candida albicans) biofilm on catheter material
Source: PLoS One. 2023 Jul 14;18(7):e0288452. doi: 10.1371/journal.pone.0288452 (PMC10348565; doi:10.1371/journal.pone.0288452)
Supplement: Attachment — Submitted filename: Response to reviewers.doc [file pone.0288452.s003.doc]

1. Please ensure that your manuscript meets PLOS ONE's style requirements, including those for file naming. The PLOS ONE style templates can be found at 
<https://journals.plos.org/plosone/s/file?id=wjVg/PLOSOne_formatting_sample_main_body.pdf> and 
<https://journals.plos.org/plosone/s/file?id=ba62/PLOSOne_formatting_sample_title_authors_affiliations.pdf>

According to the editor’s comment, we revised the manuscript based on the PLOS ONE style templates.

2. Thank you for stating in your Funding Statement: 
"This study was supported in part by the Research Program on Emerging and Re-Emerging Infectious Diseases of the Japan Agency for Medical Research and Development (grant number JP23fk0108679h0401 to TS)."

Please provide an amended statement that declares *all* the funding or sources of support (whether external or internal to your organization) received during this study, as detailed online in our guide for authors at <http://journals.plos.org/plosone/s/submit-now>.  Please also include the statement “There was no additional external funding received for this study.” in your updated Funding Statement. 
Please include your amended Funding Statement within your cover letter. We will change the online submission form on your behalf.

According to the editor’s comment, the Funding Statement was included in the cover letter.

This study was supported in part by the Research Program on Emerging and Re-Emerging Infectious Diseases of the Japan Agency for Medical Research and Development (grant number JP23fk0108679h0401 to TS) and for JSPS KAKENHI grant number JP23K06141 (Scientific Research (C) to Y.M.). The funders had no role in the study design, data collection, data analysis, decision to publish, or preparation of the manuscript. There was no additional external funding received for this study.

3. We note that Figures 1, 3, 4, 6 and 7 in your submission contain copyrighted images. All PLOS content is published under the Creative Commons Attribution License (CC BY 4.0), which means that the manuscript, images, and Supporting Information files will be freely available online, and any third party is permitted to access, download, copy, distribute, and use these materials in any way, even commercially, with proper attribution. For more information, see our copyright guidelines: <http://journals.plos.org/plosone/s/licenses-and-copyright>.
 
We require you to either (1) present written permission from the copyright holder to publish these figures specifically under the CC BY 4.0 license, or (2) remove the figures from your submission:
 
a. You may seek permission from the original copyright holder of Figures 1, 3, 4, 6 and 7 to publish the content specifically under the CC BY 4.0 license. 
 
We recommend that you contact the original copyright holder with the Content Permission Form (<http://journals.plos.org/plosone/s/file?id=7c09/content-permission-form.pdf>) and the following text:
“I request permission for the open-access journal PLOS ONE to publish XXX under the Creative Commons Attribution License (CCAL) CC BY 4.0 (<http://creativecommons.org/licenses/by/4.0/>). Please be aware that this license allows unrestricted use and distribution, even commercially, by third parties. Please reply and provide explicit written permission to publish XXX under a CC BY license and complete the attached form.”

Please upload the completed Content Permission Form or other proof of granted permissions as an ""Other"" file with your submission. 
 
In the figure caption of the copyrighted figure, please include the following text: “Reprinted from [ref] under a CC BY license, with permission from [name of publisher], original copyright [original copyright year].”
 
b. If you are unable to obtain permission from the original copyright holder to publish these figures under the CC BY 4.0 license or if the copyright holder’s requirements are incompatible with the CC BY 4.0 license, please either i) remove the figure or ii) supply a replacement figure that complies with the CC BY 4.0 license. Please check copyright information on all replacement figures and update the figure caption with source information. If applicable, please specify in the figure caption text when a figure is similar but not identical to the original image and is therefore for illustrative purposes only.

The image of silkworm was produced by me and firstly published in PLoS One (Matsumoto Y., et al., PLoS One, 2011). According to the editor’s comment, we added the Content Permission Form.

[Reference]

Matsumoto Y, Sumiya E, Sugita T, Sekimizu K. [An invertebrate hyperglycemic model for the identification of anti-diabetic drugs.](https://pubmed.ncbi.nlm.nih.gov/21479175/) PLoS One. 2011 6:e18292. doi: 10.1371/journal.pone.0018292.

Reviewer #1: In the manuscript entitled “Silkworm model of biofilm formation: In vivo evaluation of antimicrobial tolerance of a cross-kingdom dual-species (Escherichia coli and Candida albicans) biofilm on catheter material”, the authors evaluate the antimicrobial tolerance of Escherichia coli and Candida albicans biofilm on catheter material. The experiments have been performed well and the manuscript is good, but requires minor revision before considering for publication in PLOS ONE. The comments for the authors are mentioned below.

1. In some places, grammar and English have to be improved. Use standard tools to correct the same.

Following the referee’s comment, the revised manuscript was edited by professional native English-speaking science editors (SciTechEdit International, LLC, CO, USA).

2. Include ethical clearance certificate

Following the referee’s comment, we added the file of the ethical clearance certificate.

3. Give justification that 1h is enough as the incubation time for MEPM. Was the experiment performed for other time durations?

In an experiment with silkworms 6 h after the administration of MEPM, some silkworms died in the saline group as the control. In addition, the half-life of MEPM in humans after reaching its maximum blood concentration is approximately 1 (Johan W., et al. Clinical Pharmacokinetics, 1995). Therefore, we examined one hour after inoculation for evaluating the effects of MEPM.

[Reference]

Johan W. Mouton & John N. van den Anker. Meropenem Clinical Pharmacokinetics. Clinical Pharmacokinetics. 1995 28:275–286.

4. Check the results and discussion section thoroughly. Some of the places and methods are mentioned which have to be included only in the methods section.

According to the reviewer’s comment, we changed the places of the sentences appropriately in the revised manuscript.

Moreover, the following sentences have been removed from the Results section of the revised manuscript.

・These results suggest *E. coli* (2 × 10^8^ cells/larva) inoculated into PF-inserted silkworms formed a biofilm on the PF surface in the silkworms.

・These results suggest that the dual-species biofilm formed by *E. coli* and *C. albicans* on the PF surface in the silkworms does not inhibit the growth of either organism.

・These results suggest that *C. albicans* in a dual-species biofilm with *E. coli* promotes the MEPM tolerance of *E. coli* in silkworms.

・These results suggest that MEPM tolerance of *E. coli* in a dual-species biofilm are not suppressed by co-administration with the antifungal drug MCFG.

5. Include the following ref in the manuscript
DOI: 10.1111/jam.15402
DOI: 10.1007/s11814-021-1054-3
DOI: 10.1002/pat.5753
DOI: 10.3390/antibiotics11050573

According to the reviewer’s comment, we added the references in the Discussion section of the revised manuscript (Page 20, lines 334-341).

[Page 20, lines 334-341]

Recently, techniques have been developed for high-throughput measurement of biofilm formation by *Candida* spp. using microplates (Sadanandan B., et al., Journal of Applied Microbiology, 2021, Sadanandan B., et al., Korean J. Chem Eng., 2022). Based on these techniques, a method has been established to measure polymicrobial biofilm formation by *C. albicans* and *E. coli* (Ashrit P., et al., Polymers Advanced Technologies, 2022). Moreover, the method can be used to identify samples that inhibit the polymicrobial biofilm formation of *C. albicans* and *E. coli* (Ashrit P., et al., Antibiotics, 2022). Therefore, candidate compounds that inhibit the polymicrobial biofilm formation of *C. albicans* and *E. coli* can be obtained in future *in vitro* tests. We assumed that the effectiveness of the candidate compounds *in vivo* could be evaluated using the silkworm model developed in this study.

[References]

・Sadanandan B, Vaniyamparambath V, Lokesh KN, Shetty K, Joglekar AP, Ashrit P, Hemanth B. *Candida albicans* biofilm formation and growth optimization for functional studies using response surface methodology. J Appl Microbiol. 2022 132:3277-3292. doi: 10.1111/jam.15402.

・Sadanandan B, Ashrit P, Nataraj LK, Shetty K, Jogalekar AP, Vaniyamparambath V, Hemanth B. High throughput comparative assessment of biofilm formation of *Candida glabrata* on polystyrene material. Korean J Chem Eng. 2022 39:1277-1286.

・Ashrit P, Sadanandan B, Nataraj LK, Shetty K, Vaniyamparambath V, Raghu AV. A microplate-based Response Surface Methodology model for growth optimization and biofilm formation on polystyrene polymeric material in a *Candida albicans* and *Escherichia coli* co-culture. Polymers for Advanced Technologies. 2022 33:2872-2885.

・Ashrit P, Sadanandan B, Shetty K, Vaniyamparambath V. Polymicrobial Biofilm Dynamics of Multidrug-Resistant *Candida albicans* and Ampicillin-Resistant *Escherichia coli* and Antimicrobial Inhibition by Aqueous Garlic Extract. Antibiotics (Basel). 2022 11:573. doi: 10.3390/antibiotics11050573.

Reviewer #2: The work has its merits especially as an in vivo silkworm model has been developed to study E. coli and its co culture with Candida albicans. However, to make the study more robust the following may be considered.

1. The authors mention that the planktonic E. coli culture was sensitive to Meropenem (MEPN) as per the previous report. What was the MIC?
Did the authors confirm the susceptibility to the antibiotic during the course of this work? If so, please include an image here. A simple agar disc diffusion experiment will clearly demonstrate the sensitivity or resistance of E. coli and C. albicans at different concentrations of the antimicrobials.

According to the reviewer’s comment, we determined the MIC value of MEPM against *E. coli* RB-3. Susceptibility testing for meropenem (MEPM) was performed using the MicroScan AST panel (Beckman Coulter, Pasadena, CA, USA) according to CLSI M100-Ed31. The MIC value of MEPM was less than 1 µg/mL. The result was described in Supplementary Table 1 (S1 Table) of the revised manuscript (Page 19, line 308). Because the fungus *C. albicans* is not sensitive to the antibacterial drug MEPM, observation of the inhibitory circle by the disc diffusion experiment is difficult on plates with *C. albicans* and *E. coli*.

[Reference]

Clinical and Laboratory Standards Institute (CLSI). Performance standards for antimicrobial susceptibility testing; M100-Ed30. 30th ed. Wayne, PA: CLSI; 2020.

2. The silkworms have been reared on a diet containing antibiotics, please mention them. Also have you maintained a control set of silk worms that were fed a diet without antibiotics just to rule out any antimicrobial resistance conferred thereby.

Following the reviewer’s comment, we added the description that the silkworms reared on a diet containing antibiotics in the Materials & Methods section of the revised manuscript (Page 6, lines 99-100). The artificial diet without antibiotics was fed 24 hours before the infection experiments (Page 6, lines 100-101).

[Page 6, lines 99-101]

Silkworms were fed an artificial diet, Silkmate 2S, containing antibiotics purchased from Ehime-Sanshu Co., Ltd. (Ehime, Japan). Fifth-instar silkworms were fed with an antibiotic-free artificial diet (Sysmex Corporation, Hyogo, Japan) for 1 day.

3. Please justify the choice of PF as the prosthetic material in your study.

According to the reviewer’s comment, we changed the sentence in the revised manuscript (Page 5, lines 74-77).

[Page 5, lines 74-77]

In this study, we established an experimental system for evaluating biofilm formation by *E. coli* on the surface of a polyurethane fiber (PF) inserted in a silkworm and investigated the effect of MEPM against a dual-species biofilm with *C. albicans*. Polyurethane is a central venous catheter material (Ju DB., et al., Tissue Eng Regen Med. 2022).

[Reference]

Ju DB, Lee JC, Hwang SK, Cho CS, Kim HJ. Progress of Polysaccharide-Contained Polyurethanes for Biomedical Applications. Tissue Eng Regen Med. 2022 19(5):891-912. doi: 10.1007/s13770-022-00464-2.

4. Please include the CFU plate images. This is crucial as one of the major claims is about the increased tolerance of E. coli to MEPN in the presence of 106 C. albicans cells as compared to 105 cells.

According to the reviewer’s comment, we added the plate images in the Supplementary Fig. 1 (S1 Fig) of the revised manuscript (Page 17, line 281). In this study, viable *E. coli* cells on the surface of the PFs in the silkworms were grown on nutrient agar medium containing micafungin (1 μg/mL), which inhibits the growth of *C. albicans*. The sentences were described in the Materials & Methods section of the revised manuscript (Page 8, lines 136-139).

[Page 8, lines 136-139]

The CFUs of *E. coli* and *C. albicans* were calculated: 1 μg/mL micafungin was added to nutrient agar medium (to selectively grow only *E. coli*) or 100 μg/mL streptomycin was added to Sabouraud agar medium (to selectively grow only *C. albicans*). *E. coli* RB-3 and *C. albicans* SC5314 strains were susceptible to streptomycin and micafungin, respectively.

5. The claim about increased tolerance should be supported with broth microdilution wherein growth OD & MTT assay could be performed.

Because OD and MTT assays cannot distinguish between the survival of *E. coli* and *C. albicans*, we assessed each by measuring their respective colony counts in this study. We used media selective for each organism, such as media containing antifungal drug micafungin where *E. coli* proliferates selectively, and media containing antibacterial drug streptomycin where *C. albicans* grows selectively, to measure the colony counts. We added the sentences to clarify this point in the Materials & Methods section of the revised manuscript (Page 8, lines 136-139).

[Page 8, lines 136-139]

The CFUs of *E. coli* and *C. albicans* were calculated: 1 μg/mL micafungin was added to nutrient agar medium (to selectively grow only *E. coli*) or 100 μg/mL streptomycin was added to Sabouraud agar medium (to selectively grow only *C. albicans*). *E. coli* RB-3 and *C. albicans* SC5314 strains were susceptible to streptomycin and micafungin, respectively.

6. Please include the CFU results for concentrations below 105 and above 106 C. albicans cells.

We performed the dose response of *C. albicans* cells on the tolerance to MEPM of *E.coli*. The addition of *C. albicans* (10^4^-10^5^ cells) did not increase the tolerance of MEPM of *E. coli*. The result is shown in Fig. 6C of the revised manuscript.

7. Crystal violet (CV) stained Light microscopic images can be strengthened with fluorescence microscopy or SEM/CLSM. Also please mention the magnification of the bright field microscopic images.

According to the reviewer’s comment, we added the below sentences in the revised manuscript (Pages 20-21, lines 341-344). Moreover, we added the magnification of the bright field microscopic images on the Fig. 1B and 4B in the revised manuscript.

[Pages 20-21, lines 341-344]

Biofilm clarification with transparency technology has enabled the highly sensitive observation of biofilms using fluorescence microscopy (Sugimoto S et al., Communications Biology, 2023). In the future, the development of high-resolution observation methods that incorporate analysis using such technologies will be an important subject.

[Reference]

Sugimoto S, Kinjo Y. Instantaneous Clearing of Biofilm (iCBiofilm): an optical approach to revisit bacterial and fungal biofilm imaging.

Commun Biol. 2023 6:38. doi: 10.1038/s42003-022-04396-4.

8. As per published reports E. coli controls C. albicans growth however, as per latest reports C. albicans overcomes this inhibition at 24h. In light of this have you carried out the CFU experiment at different time points to understand the relationship dynamics in dual cultures which in turn will influence the susceptibility or resistance to drugs. Please include this perspective in your discussion as well.

According to the reviewer’s comment, we added the sentences in the Discussion section of the revised manuscript (Pages 17-18, lines 288-291). We assumed that *E. coli* affects the growth of *C. albicans* at the early time point.

[Pages 17-18, lines 288-291]

In this study, we investigated biofilm formation at 18 h after inoculation with *E. coli* and *C. albicans*. In an *in vitro* experiment, *E. coli* inhibits the growth of *C. albicans* (Cabral DJ et. al., Microb Cell, 2018). We assumed that prolonged co-infection in the host environment was necessary for the formation of a dual-species biofilm for tolerance to MEPM.

[Reference]

Cabral DJ, Penumutchu S, Norris C, Morones-Ramirez JR, Belenky P. Microbial competition between *Escherichia coli* and *Candida albicans* reveals a soluble fungicidal factor. Microb Cell. 2018 5:249-255. doi: 10.15698/mic2018.05.631.

9. In vitro models for optimization of growth of Candida and E. coli individual and cocultures have been developed on several prosthetic materials. Have you optimized the conditions for growth of the culture on polyurethane foam?

We optimized the conditions for *C. albicans* SC5314 strain to form biofilms on the surface of polyurethane fibers *in vitro* and *in vivo* using silkworms (Matsumoto Y., et al., Med. Mycol., 2021). Furthermore, we optimized biofilm formation by co-cultivation of the *C. albicans* SC5314 and *E. coli* RB-3 strains used in this study *in vitro* (Eshima S., et al., Microorganisms, 2022). Based on the information, we further optimized the amount of *E. coli* or *C. albicans* administered to silkworms in this study.

[References]

・Matsumoto Y, Kurakado S, Sugita T. Evaluating *Candida albicans* biofilm formation in silkworms.

Med Mycol. 2021 59:201-205. doi: 10.1093/mmy/myaa064.

・Eshima S, Kurakado S, Matsumoto Y, Kudo T, Sugita T. *Candida albicans* Promotes the Antimicrobial Tolerance of *Escherichia coli* in a Cross-Kingdom Dual-Species Biofilm.

Microorganisms. 2022 10:2179. doi: 10.3390/microorganisms10112179.

10. Please compare the merits of your in vivo model with that of published in vitro models.

According to the reviewer’s comment, we added the sentences in the Discussion section of the revised manuscript (Page 21, lines 349-352).

[Page 21, lines 349-352]

The *in vivo* biofilm infection model using silkworms, which can be used to evaluate the effects of host factors and pharmacokinetics of antimicrobial drugs, might contribute to the development of new treatment strategies for device-associated dual-species biofilm infections.
