# Supplementary material for: Silkworm model of biofilm formation: In vivo evaluation of antimicrobial tolerance of a cross-kingdom dual-species (Escherichia coli and Candida albicans) biofilm on catheter material
Source: PLoS One. 2023 Jul 14;18(7):e0288452. doi: 10.1371/journal.pone.0288452 (PMC10348565; doi:10.1371/journal.pone.0288452)
Supplement: Attachment — Submitted filename: Response to the reviewers comments.doc [file pone.0288452.s004.doc]

Reviewer #1: The manuscript entitled “Silkworm model of biofilm formation: In vivo evaluation of antimicrobial tolerance of a cross-kingdom dual-species (Escherichia coli and Candida albicans) biofilm on catheter material”, the authors evaluate the antimicrobial tolerance of Escherichia coli and Candida albicans biofilm on catheter material. The experiments have been performed well and the manuscript is good.

Thank you very much for the time spent reviewing our manuscript.

Reviewer #2: Review Comments
1. The Microscan AST in place of agar disc diffusion carried out for E. coli is acceptable.
Candida albicans sensitivity or resistance to the antimicrobial was meant for the relevant antimicrobial micafungin & not Meropenem (MEPM), therefore the term antimicrobials had been used, so please include the MIC information for Candida as well.

According to the reviewer’s comment, we determined MIC value of micafungin against *Candida albicans* SC5314. The result was described in Supplementary Table S2. Furthermore, we described the below sentence in the Discussion section of the revised manuscript (Page 20, lines 329-330).

[Page 20, lines 329-330]

The *C. albicans* SC5314 strain exhibited sensitivity to MCFG in an *in vitro* drug susceptibility test (S2 Table in S1 File).

2. The antibiotics present in the silkworm artificial diet have not been included so please include them.

According to the reviewer’s comment, we described the below sentence in the Materials and Methods section of the revised manuscript (Page 6, lines 99-100). Since vancomycin is not absorbed from intestinal tract to hemolymph, we assumed that diet containing vancomycin does not affect the infection experiments with *E. coli*.

[Page 6, lines 99-100]

Silkworms were fed an artificial diet, Silkmate 2S (Ehime-Sanshu Co., Ltd., Ehime, Japan) mixed with vancomycin (300 µg/g of Silkmate 2S).

3. Experiments on Growth OD & MTT were suggested as in the earlier manuscript version MIC was not included. Now that Microscan AST has been done to determine MIC of MEPM against E. coli it is OK. However as indicated in point 1 the MIC information for Candida should also be included.

According to the reviewer’s comment, we determined MIC value of micafungin against *Candida albicans* SC5314. The result was described in Supplementary Table S2. Furthermore, we described the below sentence in the Discussion section of the revised manuscript (Page 20, lines 329-330).

[Page 20, lines 329-330]

The *C. albicans* SC5314 strain exhibited sensitivity to MCFG in an *in vitro* drug susceptibility test (S2 Table in S1 File).

Thank you very much for the time spent reviewing our manuscript.
